# PEAK1 promotes invasion and metastasis and confers drug resistance in breast cancer

**DOI:** 10.1007/s10238-021-00761-5

**Published:** 2021-09-23

**Authors:** Xingang Wang, Yan Zheng, Yu Wang

**Affiliations:** 1 Department of Breast Surgery, The Affiliated Hospital of Qingdao University, Qingdao, 266003 Shandong China; 2 Department of Operating Room, The Affiliated Hospital of Qingdao University, Qingdao, 266003 Shandong China

**Keywords:** Breast cancer, Chemotherapy resistance, Metastasis, Pseudopodium-enriched atypical kinase 1

## Abstract

Pseudopodium-enriched atypical kinase 1 (PEAK1) has been reported to be upregulated in human malignancies and is correlated with a poor prognosis. Enhanced PEAK1 expression facilitates tumor cell survival, invasion, metastasis and chemoresistance. However, the role of PEAK1 in breast cancer is unclear. We investigated PEAK1 expression in breast cancer and analyzed the relationship with clinicopathological status and chemotherapy resistance. We also investigated the role of PEAK1 in breast cancer cells in vitro and in vivo. Immunohistochemistry for PEAK1 was performed in 112 surgically resected breast cancer tissues. The association between clinicopathological status, chemotherapy resistance and PEAK1 expression was determined. The effect of PEAK1 overexpression or downregulation on proliferation, colony formation, invasion, migration, metastasis and doxorubicin sensitivity in MCF-7 cells in vitro and in vivo was studied. PEAK1 was overexpressed in breast cancer tissues. High PEAK1 expression was correlated with tumor size, high tumor grade, tumor stage, lymph node metastasis, recurrence, Ki-67 expression, Her-2 expression and chemotherapy resistance. Inhibiting PEAK1 decreased cell growth, invasion, metastasis and reversed chemoresistance to doxorubicin in breast cancer cells both in vitro and in vivo. High PEAK1 expression was associated with the invasion, metastasis and chemoresistance of breast cancers. Furthermore, targeting PEAK1 inhibited cell growth and metastasis and reversed chemoresistance in breast cancer cells. Targeting PEAK1 could be an effective treatment strategy for breast cancer.

## Introduction

Breast cancer metastasis results in poor patient prognosis and increased mortality, and the mechanisms linking metastasis with decreased overall prognosis have yet to be fully understood. Tumor resection combined with radiotherapy, endocrine therapy and chemotherapy is the main approach to treat breast cancer. However, the development of chemoresistance limits the effectiveness of chemotherapy [1]. Therefore, identifying the molecular mechanisms contributing to breast cancer progression and chemoresistance could provide novel biomarkers for the precise prediction of patient prognosis and for molecular targeted therapy.

Overexpression of Pseudopodium enriched atypical kinase 1 (PEAK1/Sgk269), a protein discovered in the pseudopodia of migrating cells [2], is also found in colorectal cancer [3] and pancreatic cancer [4]. PEAK1 expression is associated with metastasis and proliferation in many cancer types, such as colorectal cancer [3, 5], lung cancer [6] and pancreatic cancer [4]. Forced PEAK1 expression can cause cell cycle deregulation and resistance to chemotherapy in pancreatic cancer cells [4]. Altering PEAK1 expression can interfere with tumor formation and metastasis in pancreatic cancer cells in vivo, indicating that PEAK1 plays an important role in pancreatic cancer growth and metastasis. In breast cancer cells in vivo, forced PEAK1 expression can induce new blood vessel formation by upregulating vascular endothelial growth factor receptor-2, which facilitates cell movement and growth [7].

It has recently been found that PEAK1 overexpression is significantly associated with advanced clinical stage and poor prognosis in colon cancer [3] and pancreatic cancer [4]. In breast cancer, PEAK1 levels correlate with mesenchymal cell gene expression, poor cellular differentiation and disease relapse [8]. Croucher et al. [9] reported that PEAK1 overexpression was detected in a subset of basal, HER2-positive and luminal breast cancers. However, the relationship between PEAK1 expression and clinicopathological status and the relationship between PEAK1 expression and chemosensitivity in breast cancer are currently unknown.

In the present study, we examined PEAK1 protein expression in human breast cancer tissues and explored the relationship between PEAK1 expression and clinical characteristics. Furthermore, we investigated the effect of PEAK1 on breast cancer cell growth, invasion, migration, metastasis and doxorubicin sensitivity in vitro and in vivo.

## Materials and methods

### Cell culture

The human breast cancer cell line MCF-7 was purchased from the Institute of Cell Research, Chinese Academy of Sciences (Beijing, China). Doxorubicin (DOX)-resistant MCF-7 cells (MCF-7^DOX^) were established by applying increasing concentrations of DOX (0.1–2 μg/mL) to MCF-7 cells in vitro. Cells were cultured in DMEM (Sigma) supplemented with 10% FBS (Gibco), 100 units/ml of penicillin and 100 μg/ml streptomycin in a 5% CO_2_ chamber at 37 °C. MCF-7^DOX^ cells were cultured at a final concentration of 2.0 μg/mL DOX.

### Patient samples

Breast cancer samples were obtained from the Breast Disease Center, at The Affiliated Hospital of Qingdao University. In total, 112 surgically resected tumors from Feb. 2012–2018 were included. Pathological diagnosis was verified by two pathologists independently. All human samples were collected with informed consent from the patients according to the International Ethical Guidelines for Biomedical Research Involving Human Subjects. The study was performed after approval by the Institutional Review Board of the Affiliated Hospital of Qingdao University. Written informed consent was obtained from each patient.

### Immunohistochemistry

Formalin-fixed paraffin-embedded tissue sections from excised specimens were processed for immunohistochemistry (IHC) according to standard procedures. Specific primary antibody against PEAK1 was purchased from Novusbio( No:nbp1-91,052,1:220; Beijing China); specific primary antibodies hormone receptors (estrogen receptor (ER) and progesterone receptor (PR)), human epidermal growth factor receptor-2 (HER2) and Ki-67 were purchased from Sigma-Aldrich (Shanghai, China). IHC staining was performed according to the manufacturer’s instructions**.** The expression of PEAK1 was positive when 10% of tumor cells showed PEAK1 immunopositivity, and negative when less than 10% of tumor cells showed PEAK1 immunopositivity. The fraction of proliferating cells (Ki-67-positive) was based on a count of at least 500 tumor cells in the peripheral area, including the hot spot. The cell count for each image was performed manually using ImageJ software (National Institutes of Health, USA). In each case, the Ki-67 values were presented as the percentage of positive cells. The median value for Ki67 was 15% (range: < 1%—98%), which was used to define low or high Ki-67 expression levels: Ki-67 values < 15% were defined as “low Ki-67,” whereas values ≥ 15% were defined as “high Ki-67.” At present, the cutoff between “high” and “low” values for Ki-67 varies between laboratories, as well as between times. Initially, a level of < 14% was defined as “low” value for Ki-67 in St Gallen consensus in 2011 [10]. Subsequently, the majority of the St Gallen panel voted that a threshold of < 20% was indicative of “low” Ki-67 status in 2013 [11]. However, we could not determine which one, either 14 or 20%, is a better cutoff for Ki-67 yet. In this study, we chose the median value of 15% for Ki-67 as the threshold. The positive cell rates for the hormone receptors were determined by IHC, and a value of ≥ 1% was considered positive [12]. Tumors with a HER2 3 + score on IHC were considered HER2-positive. If the HER2 score was 2 + , we performed an immunofluorescence in situ (FISH) assay to further evaluate the HER2 amplification status.

### PEAK1 shRNA vector construction and transfection

Short hairpin RNAs directed against the human PEAK1 gene (PEAK1 shRNA) were synthesized and cloned into the pcDNA3.1 expression vector (Shanghai, China) according to the manufacturer’s instructions. The constructed vectors (PEAK1 shRNA or NC shRNA) were transfected into MCF-7 cells using Lipofectamine 3000 reagent (Invitrogen, Shanghai, China) according to the manufacturer’s instructions. PEAK1 shRNA- or NC shRNA-transfected MCF-7 cells were selected using puromycin (10 mg/ml) for five days. Puromycin-resistant colonies were then single-cell cloned and expanded. Relative PEAK1 protein was detected by western blotting.

### Overexpression plasmid constructs and transfection

An adenoviral vector expressing full-length human PEAK1 was constructed using the AdEasy Adenoviral Vector System according to the manufacturer’s instructions. Viral particles were produced by GenScript Biotechnology, China. Viral particles containing PEAK1 overexpressing vectors or control vectors were used to infect MCF-7 cells. Transfected cells were selected with G418 (600 μg/ml, Gibco) for 10–12 days. The expression of PEAK1 in stable PEAK1-transfected colonies was detected by western blotting.

### MTT assay

Forty-eight hours after transfection of MCF-7 cells with Lv-PEAK1, PEAK1 shRNA, or the appropriate control, cells were plated (300 cells/well) in a 96-well plate in triplicate for 24 h. The cells were then exposed to a concentration of 2.0 μg/mL doxorubicin for 72 h. Subsequently, 20 μL of MTT (Sigma-Aldrich) was added to each well and then incubated for four hours at 37 °C in a 5% CO2 humidified atmosphere. The optical density at 450 nm was measured and considered an indirect index of relative cell viability.

### Colony formation assay

Forty-eight hours after MCF-7 cells were transfected with Lv-PEAK1, PEAK1 shRNA, or the appropriate controls, cells were plated in triplicate at 1000 cells/well in 6-well plates with or without 2.0 μg/mL doxorubicin. Cells were cultured for 12 days, fixed with methanol and stained with Giemsa stain (GS, Sigma-Aldrich). Colonies were then counted.

### Matrigel invasion assay

Cell migration and invasion were determined using a Transwell chamber assay (BD Biosciences) according to the manufacturer’s instructions. Twenty-four hours after MCF-7 cells (1 × 10^5^ cells) were transfected with Lv-PEAK1, PEAK1 shRNA, or the appropriate control, they were added to the upper compartment of chambers, and DMEM plus 10% FCS was added to the lower compartment. Bestatin (Sigma) was added to both compartments. Cells were incubated for 24 h, and the total number of invaded cells was calculated according to the manufacture’s instruction. Experiments were run in triplicate.

### Western blotting

Cells and tissues were lysed, and protein concentration was measured using a BCA Protein Assay Kit (Pierce). Proteins were then resolved on an SDS-PAGE gel, transferred to polyvinylidene fluoride membranes (Millipore) and probed with primary antibodies against PEAK1 and α-Tubulin. Antibody against PEAK1 was purchased from Invitrogen (NoPAS-101940,1:1000; shanghai, China). Band densitometry analysis was performed using ImageJ software (NCI).

### In vivo* metastasis assay*

Female BALB/c nude mice (5–6 weeks of age, 16–18 g) were obtained from the National Rodent Shanghai Experimental Branch Center, Chinese Academy of Sciences (Shanghai, China). All experimental procedures involving animals were conducted in accordance with the institutional guidelines by the Affiliated Hospital of Qingdao University. MCF-7 cells stably transfected with Lv-PEAK1, PEAK1 shRNA or the appropriate controls, were cultured to the log phase. A total of 2 × 10^6^ cells were injected into the tail vein of mice, with six mice per group. After five weeks, whole lung tissues were removed and the numbers of visible nodules on the lung surface were counted. Hematoxylin and eosin staining was used to evaluate tumor metastases.

### In vivo* tumor growth*

A total of 5 × 10^6^ stably transfected PEAK1 shRNA MCF-7^DOX^ or Lv-PEAK1 MCF-7 cells were injected subcutaneously into the left front flank of mice. From days 3–9, DOX was administered to all groups of mice intravenously (0.1 mL, 10 mg/kg) for a total of four times at two-day intervals. Tumor dimensions were measured in two dimensions with microcalipers every other day, and tumor volumes were calculated using the following formula: tumor volume = (length × width^2^)/2. Non-retrospective ethical approval was obtained for the animal experiments conducted in this study.

### Statistical analysis

Data are shown as mean ± SEM. Data were analyzed using the Student t-test or ANOVA with Tukey’s post hoc test, as appropriate. *P* < 0.05 was considered statistically significant.

## Results

### PEAK1 is overexpressed in breast cancer tissues

**Fig. 1:**
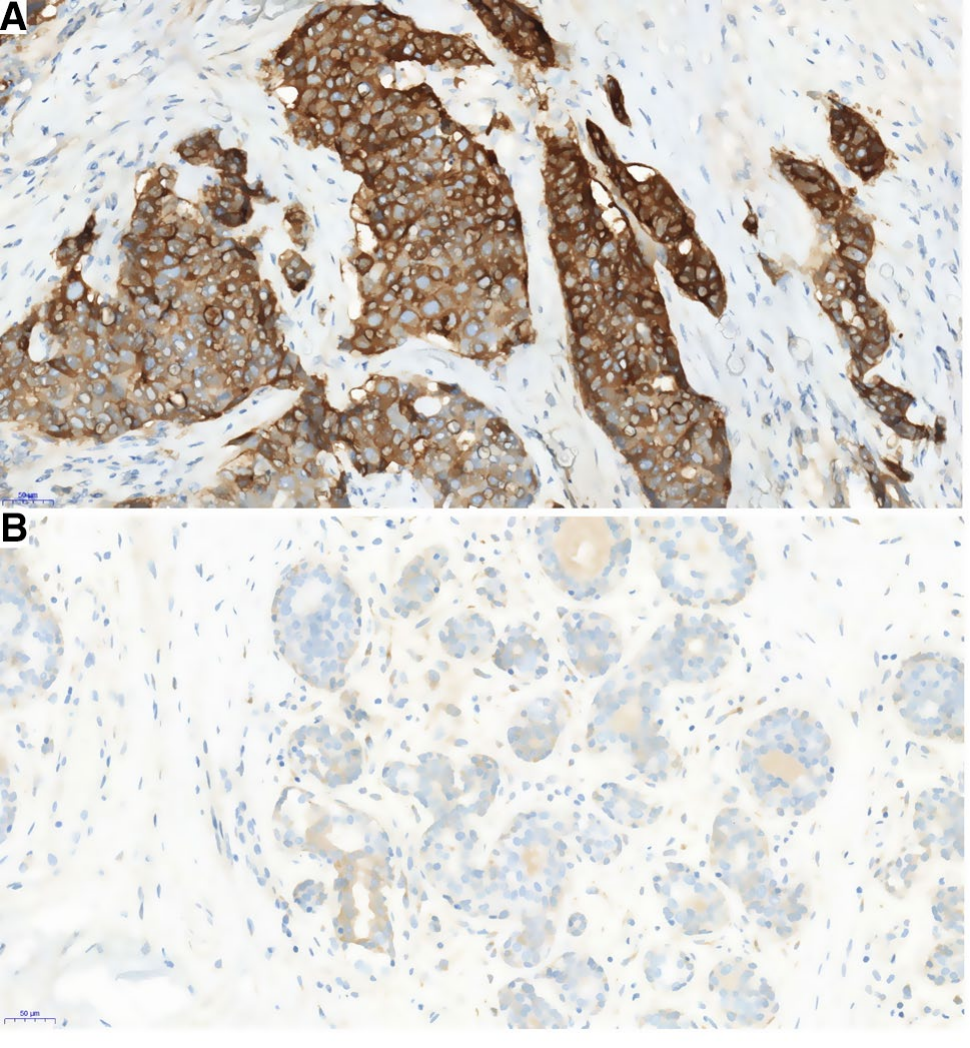
PEAK1 immunohistochemistry in breast cancer and adjacent normal tissues. **a** High PEAK1 expression in breast cancer tissues; **b** Low PEAK1 expression in the adjacent tissues of (**a**)

Using immunohistochemical methods, we measured PEAK1 expression in 112 breast cancer cases and 34 cases with corresponding adjacent normal tissues (Fig. 1). PEAK1 expression was significantly upregulated in 61.6% (69/112) of breast cancer tissues in comparison with 26.4% (9/34) adjacent normal tissues (*P* = 0.033). PEAK1 overexpression was associated with tumor size, tumor grade, tumor stage, advanced nodal status, regional recurrence, HER2, Ki-67 and chemotherapy status (Table 1).

**Table 1:** PEAK1 expression and clinicopathological status in 112 patients with breast cancer

Variable	PEAK1 expression		*P*-value
	Low expression(n = 43)	High expression(*n* = 69)	
Age(year)			0.547
≤ 50	15	28	
> 50	28	41	
Tumor size (cm)			0.032
≤ 2	27	29	
> 2	16	40	
Tumor grade			0.049
1	6	11	
2	26	26	
3	11	32	
T stage			0.012
T1	26	24	
T2	17	40	
T3	0	5	
LN metastasis			0.036
Negative	12	33	
Positive	31	36	
Distant metastasis			0.073
No	30	58	
Yes	13	11	
ER			0.074
Positive	28	33	
Negative	15	36	
PR			0.262
Positive	24	31	
Negative	19	38	
HER-2			0.0218
Positive	9	29	
Negative	34	40	
Triple-negative			0.180
Positive	9	8	
Negative	34	61	
Chemotherapy			0.0135
No	29	30	
Yes	14	39	
Radiation therapy			0.208
No	34	47	
Yes	9	22	
Endocrine therapy			0.269
No	30	41	
Yes	13	28	
Local recurrence			0.243
No	28	52	
Yes	15	17	
Regional recurrence			0.0473
No	37	48	
Yes	6	21	
Ki-67			0.0366
Positive	25	53	
Negative	18	16	

### *PEAK1 promotes cell growth, invasion and migration *in vitro

**Fig. 2:**
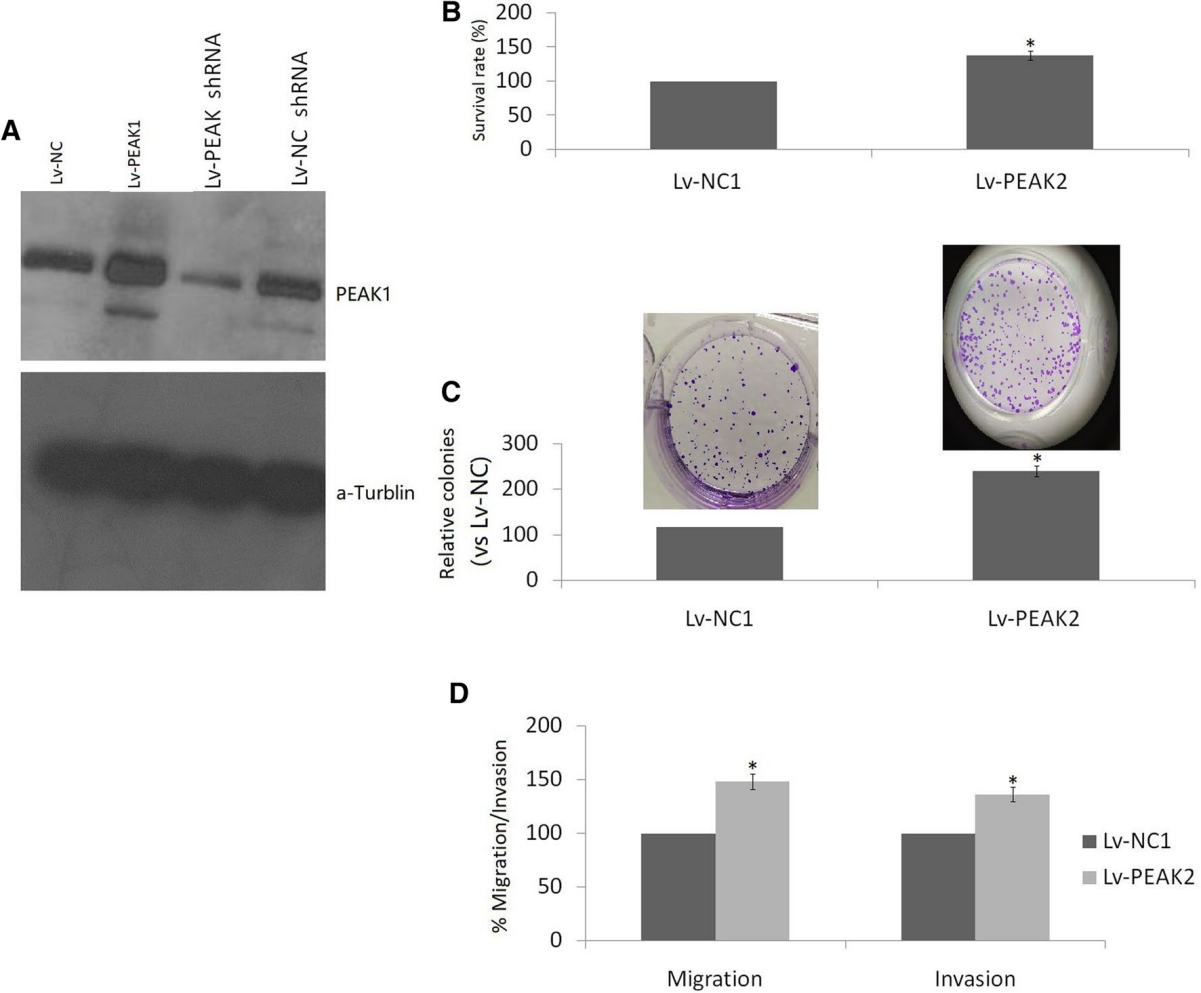
Effect of PEAK1 overexpression on cell growth, invasion and migration in vitro. **a** PEAK1 expression was measured in MCF-7/Lv-PEAK1, MCF-7/Lv-PEAK1 shRNA and MCF-7/empty vector cells by western blotting. **b** MCF-7/Lv-PEAK1 and MCF-7/empty vector cell growth was detected using the MTT assay. **c** MCF-7/Lv-PEAK1 and MCF-7/ empty vector cell growth was measured using a colony formation assay. **d** MCF-7 cell invasion and migration abilities were measured using a Transwell assay after PEAK1 was overexpressed. VS control, *P < 0.01

Lv-PEAK1 or empty vector was transfected into MCF-7 cells. PEAK1 expression was significantly increased in MCF-7/Lv-PEAK1 cells compared with MCF-7/empty vector cells (Fig. 2a). Cell growth was significantly increased in MCF-7/Lv-PEAK1 cells compared with MCF-7/empty vector cells, according to the MTT assay (Fig. 2b). To confirm the MTT data, we carried out an in vitro colony formation assay. Representative images and colony quantitation confirmed that PEAK1 overexpression significantly promotes MCF-7 cell growth (Fig. 2c).

Cell migration and invasion of transfected cells in vitro were measured using a Transwell assay. As shown in Fig. 2d, invasion and migration were significantly increased in MCF-7/Lv-PEAK1 cells compared to MCF-7/empty vector cells.

### *Targeting PEAK1 inhibits cell growth, invasion and migration *in vitro

**Fig. 3:**
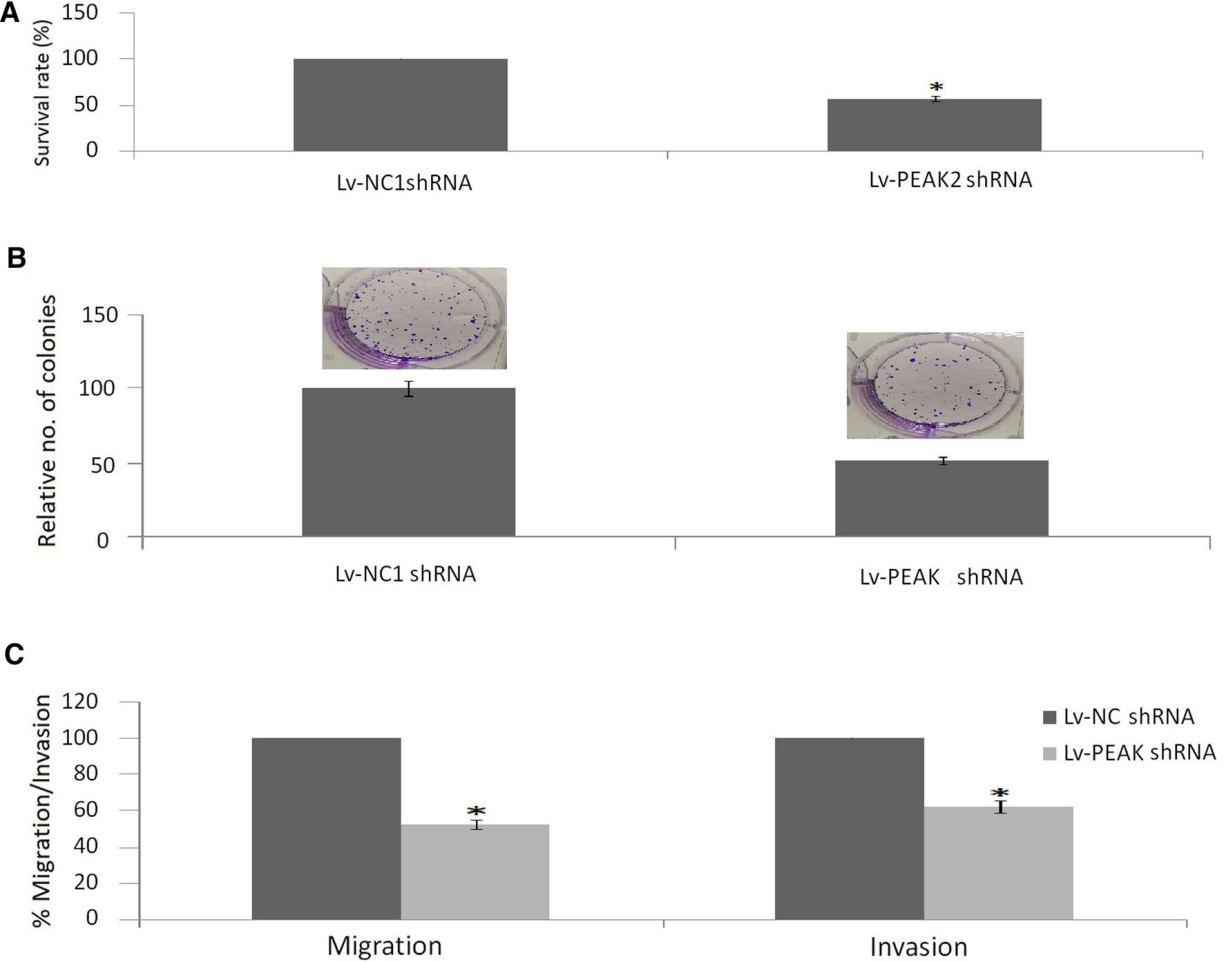
Effect of PEAK1 depletion on cell growth, invasion and migration in vitro. **a** Cell growth was measured using an MTT assay in MCF-7/Lv-PEAK1 shRNA and MCF-7/empty vector cells. **b** Cell growth was measured using a colony formation assay in MCF-7/Lv-PEAK1 shRNA and MCF-7/empty vector cells. **c** MCF-7 cells invasion and migration abilities were measured using the Transwell assay after PEAK1 depletion. VS control, *P < 0.01

Lv-PEAK1 shRNA or empty vector was transfected into MCF-7 cells. PEAK1 expression was significantly decreased in MCF-7/PEAK1 shRNA cells compared to the MCF-7/control shRNA cells (Fig. 2a). Cell growth was significantly decreased with PEAK1 depletion in MCF-7/PEAK1 shRNA cells as assessed by the MTT assay (Fig. 3a) and colony formation assay (Fig. 3b). Invasion and migration were significantly decreased in MCF-7/PEAK1 shRNA cells compared with the MCF-7/control shRNA cells (Fig. 3c).

### PEAK1 regulates doxorubicin sensitivity in MCF-7 cells

**Fig. 4:**
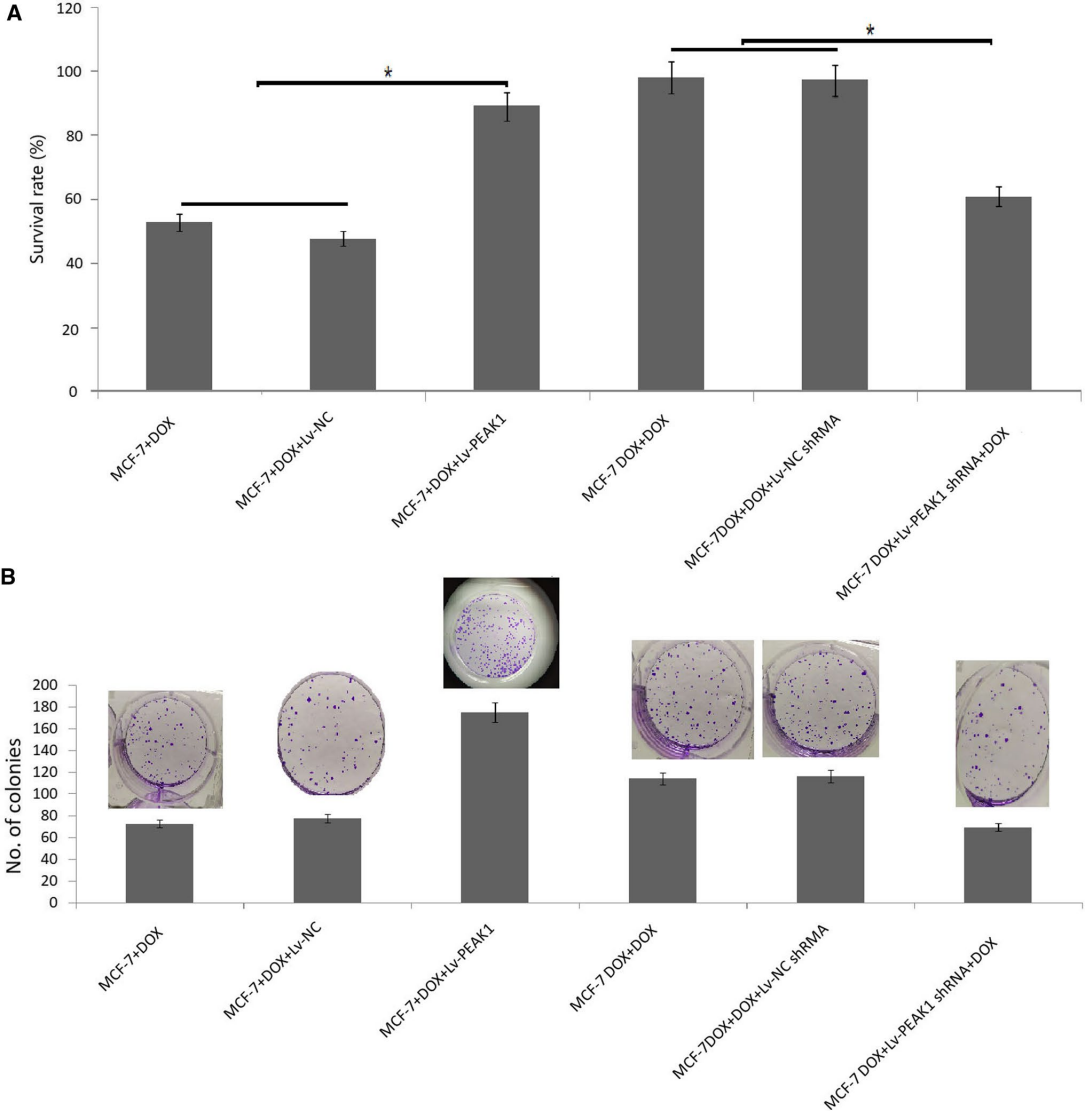
Effect of PEAK1 depletion or overexpression on doxorubicin sensitivity in MCF-7 cells. MCF-7/Lv-PEAK1, MCF-7/empty vector, MCF-7 ^DOX^/Lv-PEAK1 shRNA and MCF-7 ^DOX^/empty vector cells were treated with 2.0 μg/mL doxorubicin for 72 h. Cell growth was detected using an **a** MTT assay and **b** colony formation assay. ^*^P < 0.01

Lv-PEAK1 or empty vector was transfected into MCF-7 cells. Cells were then treated with 2.0 μg/mL doxorubicin for 72 h. PEAK1 overexpression reduced doxorubicin-induced cytotoxicity (Fig. 4a–b).

Similarly, MCF-7^DOX^ cells were transfected with PEAK1 shRNA, followed by treatment with 2.0 μg/mL doxorubicin for 72 h. PEAK1 depletion increased doxorubicin-induced cytotoxicity (Fig. 4a–b). These data suggest that targeting PEAK1 reverses doxorubicin resistance in doxorubicin-resistant breast cancer cells.

### *PEAK1 depletion increases the sensitivity of MCF-7 cells to doxorubicin *in vivo

**Fig. 5:**
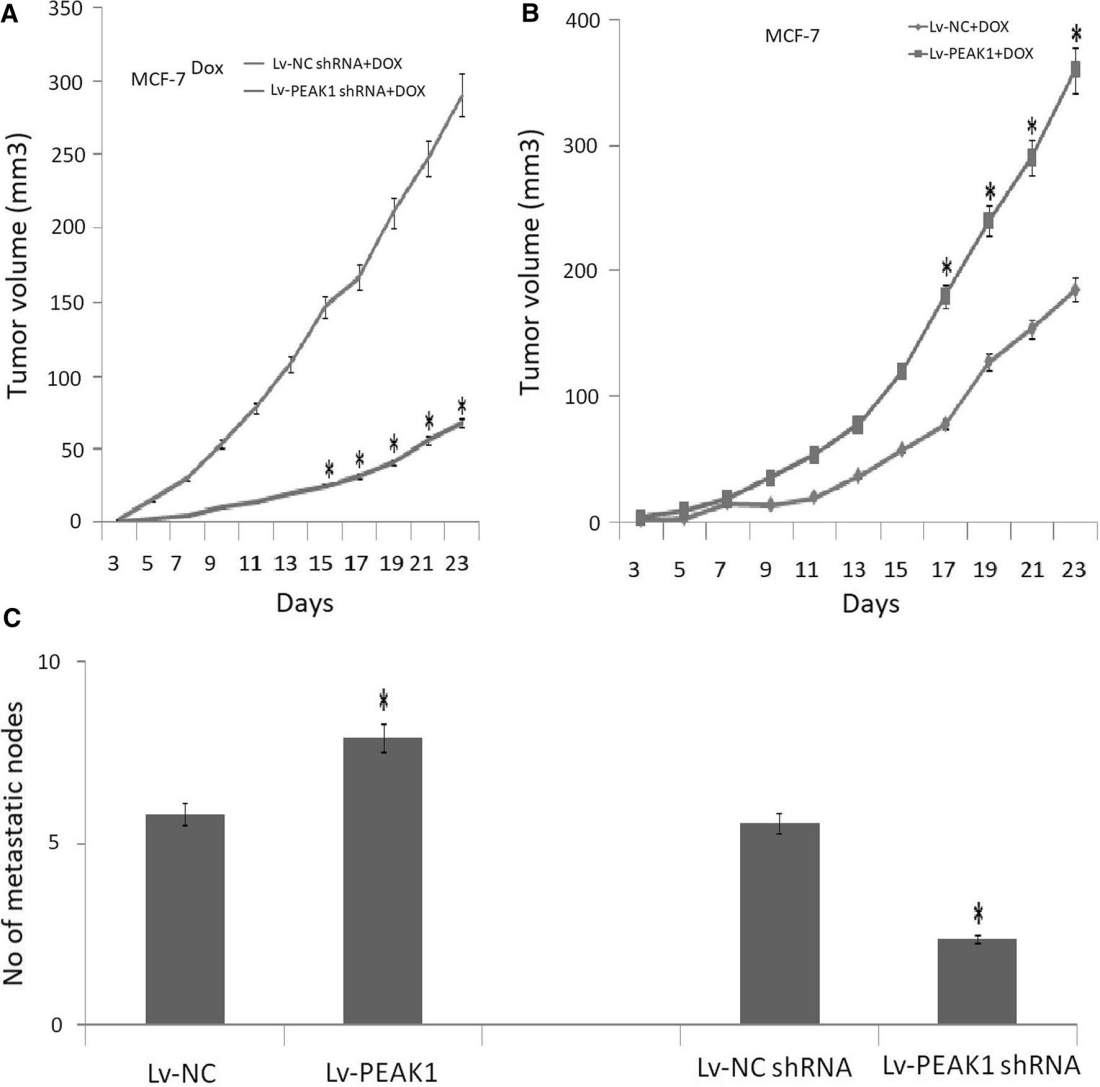
PEAK1 expression influences metastasis and chemosensitivity of MCF-7 cells in vivo. **a** MCF-7^DOX^ cells or **b** MCF-7 cells with varying PEAK1 levels were injected subcutaneously in the left front flank of female BALB/c mice (*n* = 4/group). All mice were treated with DOX by intravenous injection (days 3–9). When tumor growth became visible, tumor volume was monitored and results were graphically displayed. MCF-7/PEAK1 or NC cells, and MCF-7/Lv-NC shRNA or MCF-7/Lv-PEAK1 shRNA cells were inoculated subcutaneously into nude mice for 35 days. **c** Lung metastatic nodes were counted.*,*P* < 0.05, versus control group

To determine whether the effect of PEAK1 on in vitro chemosensitivity also is relevant to in vivo tumor growth, we injected female BALB/c mice with 5 × 10^5^ MCF-7^DOX^ or MCF-7 cells and evaluated the response to doxorubicin treatment. PEAK1 knockdown in MCF-7^DOX^ or MCF-7 cells resulted in a reduced growth rate following doxorubicin treatment (Fig. 5a–b). These data suggest that targeting PEAK1 inhibits tumor growth in vitro and sensitizes MGF-7 cells to doxorubicin treatment.

### *PEAK1 depletion inhibits lung metastasis of MCF-7 cells *in vivo

We investigated the role of PEAK1 in mediating breast cancer cell metastasis in vivo. Stably transfected PEAK1 shRNA or Lv-PEAK1 MCF-7 cells were injected into female nude mice via the tail vein. After eight weeks, the whole lung was removed and the number of visible nodules on the lung surface was counted. Hematoxylin and eosin staining was used to evaluate the tumor metastases. PEAK1 shRNA-transfected MCF-7 cells caused fewer tumor nodes (Fig. 5c), while Lv-PEAK1-transfected MCF-7 cells resulted in more tumor nodes (Fig. 5c). These data suggest that targeting PEAK1 inhibits lung metastasis in vivo.

## Discussion

Breast cancer is the most common female cancer worldwide. The propensity of breast cancer to metastasize negatively impacts therapeutic outcome. Several clinicopathological parameters with prognostic/predictive significance have been associated with metastatic suppressor expression levels.

PEAK1 is a newly described tyrosine kinase and scaffold protein that transmits integrin-mediated extracellular matrix signals to facilitate cell movement and growth. PEAK1 has been reported to be upregulated in human malignancies and has been correlated with poor prognosis [3, 4]. In the present study, we performed IHC for PEAK1 in 112 surgically resected breast cancer tissues and 43 adjacent non-tumor breast tissues. PEAK1 was localized to the cytoplasm, membrane and nucleus, with predominant cytoplasm staining. These observations were consistent with IHC staining results in colorectal cancer [3] and pancreatic cancer [4]. We also found that PEAK1 is overexpressed in breast cancer tissues at significantly higher levels than adjacent non-tumor breast tissues.

PEAK1 is upregulated in pancreatic cancer and has been associated with tumor invasion and metastasis [4]. However, PEAK1 is downregulated in gastric cancers, and higher PEAK1 expression was correlated with non-lymph node metastases and a good prognosis [13]. Huang et al. reported that PEAK1 is overexpressed in colorectal cancer tissues and that high PEAK1 expression predicts poor survival in colon cancer. Functionally, silencing PEAK1 inhibits cell proliferation, migration and invasion in vitro and inhibits the growth of tumor xenografts in nude mice [3]. However, Ding et al. reported that PEAK1 was frequently downregulated in colon cancer and significantly associated with tumor size, differentiation status, metastasis and clinical stage. PEAK1 overexpression suppressed colon cancer cell growth, invasion and metastasis in vitro and in vivo, whereas knockout had the opposite effects [5]. Here we found that PEAK1 overexpression was correlated with a larger tumor size, higher tumor stage, tumor grade, tumor stage, lymph node metastasis and recurrence, suggesting that PEAK1 overexpression may promote the malignant potential of breast cancer. Therefore, PEAK1 could serve as a valuable biomarker for the prediction of breast cancer invasion and could also play an important role in prognosis prediction. Ki-67 expression was reported to be positively correlated with a higher incidence of lymphovascular invasion and lymph node metastasis in breast cancer [14, 15]. In the current study, PEAK1 expression was correlated with lymph node metastasis and Ki-67 expression, further confirming that PEAK1 may be used as a diagnostic marker for breast cancer invasion and prognosis. In clinical settings, low-proliferative tumors are less sensitive to chemotherapy [16]. IHC for Ki-67 assessment is currently used to estimate cell proliferation and to help guide the decision on adjuvant treatment choices and prediction of neoadjuvant treatment response in breast cancer [17]. IHC for ER, PR and HER2 assessment is also used to predict sensitivity to drugs and to determine the application and types of systemic therapy [18]. In the present study, PEAK1 expression was correlated with both Ki-67 and HER2 expression in breast cancer, suggesting that PEAK1 expression may predict chemosensitivity in breast cancer. In addition, PEAK1 was overexpressed in 112 cases of breast cancer patients treated with chemotherapy. However, the difference in PEAK1 expression with radiation and hormonal treatments was not statistically significant. To confirm our observations, we tested PEAK1 expression in 53 cases of breast cancer patients being treated with neoadjuvant chemotherapy treatment and found that PEAK1 expression was significantly enhanced in chemoresistant breast cancer.

Targeting PEAK1 expression inhibits cell proliferation and metastasis in vitro and in vivo and vice versa [2–5, 7, 19]. Croucher et al. reported that PEAK1 is overexpressed in primary luminal, HER2, and basal breast cancers and cell lines as measured by Western blotting [9]. Furthermore, overexpression of PEAK1 promoted acinar growth and cell invasion in vitro. Abu-Thuraia et al. reported that PEAK1 is required for both tumor growth and metastasis in a triple negative breast cancer cell model [20]. In the present study, the blockade of PEAK1 expression inhibited cell growth, invasion and migration in vitro, and inhibited tumor growth and lung metastasis in vivo, suggesting that PEAK1 is the target gene for breast cancer gene therapy.

To further confirm whether PEAK1 expression regulates the chemosensitivity of breast cancer cells to therapeutic drugs, PEAK1 knockdown and overexpressing MCF-7 cells were treated with doxorubicin. Unsurprisingly, decreased cell and tumor growth was observed in MCF-7 cells with suppressed PEAK1 expression, whereas cell growth was increased in MCF-7 cells overexpressing PEAK1 in vitro and in vivo. These data indicate that enhanced PEAK1 expression promotes the development of chemoresistance, and vice versa.

## Conclusion

In conclusion, PEAK1 is overexpressed in breast cancer and chemoresistant breast cancers. PEAK1 overexpression is correlated with clinicopathological parameters. Measuring PEAK1 could be utilized to predict chemoresistance, and targeting PEAK1 could be used for reversing chemoresistance in breast cancer. PEAK1 may be a useful prognostic biomarker and a potential therapeutic target for patients with breast cancer.

## Data Availability

Data sharing is not applicable to this article as no new data were created or analyzed in this study.
